# From awareness to action: behavioral change is associated with functional gains and injury reduction in elite runners—a prospective observational study

**DOI:** 10.3389/fpsyg.2026.1816142

**Published:** 2026-05-12

**Authors:** Koji Murofushi, Sho Mitomo, Yosuke Yamada, Kenji Hirohata, Hidetaka Furuya, Hiroki Katagiri, Hiroshi Akuzawa, Susumu Hara, Masakazu Ito, Takehiro Ohmi, Koji Kaneoka

**Affiliations:** 1 GENTEN Research Center, Institute of Science Tokyo, Tokyo, Japan; 2 Department of Orthopedic and Spinal Surgery, Graduate School of Medical and Dental Sciences, Institute of Science Tokyo, Tokyo, Japan; 3 Sports and Health Sciences, Graduate School of Biomedical Engineering, Tohoku University, Sendai, Japan; 4 Clinical Center for Sports Medicine and Sports Dentistry, Institute of Science Tokyo, Tokyo, Japan; 5 Department of Rehabilitation, Sonoda Third Hospital, Tokyo, Japan; 6 Tokyo Medical Institute Tokyo Spine Center, Tokyo, Japan; 7 Department of Orthopedics, Dokkyo Medical University Saitama Medical Center, Saitama, Japan; 8 School of Global Studies and Collaboration, Aoyama Gakuin University, Tokyo, Japan; 9 Faculty of Sport Sciences, Waseda University, Tokyo, Japan

**Keywords:** behavioral change, embodied cognition, long-distance runners, running-related injuries (RRIs), sports injury prevention

## Abstract

**Background:**

Running-related injuries (RRIs) are a major concern among long-distance athletes. Although body awareness is important for injury prevention, the interplay between awareness, behavioral changes, and functional outcomes has not been fully examined.

**Objective:**

To investigate how body awareness and behavioral changes, measured through the Koji Awareness (KA) self-screening system, are associated with injury incidence and functional recovery across a competitive season.

**Methods:**

Eighty male collegiate runners from a single collegiate team participated in this study, and 63 runners completed the KA test before and after the season. The incidence of injury was tracked over 6 months. Participants were categorized into groups based on retrospective post-season self-report questionnaires assessing awareness and implementation of behavioral changes. Group differences in KA scores and injury incidence were analyzed using the Fisher’s exact test, Student’s *t*-test, and Mann–Whitney U test.

**Results:**

Among the 63 included athletes, 88.9% reported increased awareness, and 65.1% reported behavioral changes. Awareness alone was not significantly associated with the incidence of RRIs (Not-awareness group: 0%, Awareness group: 12.5%, *p* = 0.419). However, behavioral change was associated with both a lower injury rate (Not-behavioral group: 22.7%, Behavioral group: 4.9%, *p* < 0.05) and significantly greater functional improvement in the lower extremities. Awareness and behavioral change were significantly correlated (*p* < 0.05).

**Conclusion:**

Although awareness alone was not significantly associated with injury incidence, it was linked to improved function and a greater likelihood of behavioral change. Given the observational design, self-reported measures, and the small number of injury events, these findings should be interpreted cautiously. Nevertheless, when body awareness is accompanied by active behavior modification, it may be relevant to injury-related outcomes. The KA screening test may serve not only as a functional assessment tool but also as a potential framework for self-regulation and resilience in performance.

## 1 Introduction

Running is one of the most accessible and widely practiced physical activities, providing substantial health benefits, including improved cardiovascular function and reduced risk of lifestyle-related diseases (Lee et al., 2014; Hespanhol Junior et al., 2015). However, running-related injuries (RRIs) remain prevalent across all competitive levels. A systematic review by Van Gent et al. (2007) reported annual injury incidence rates ranging from 19.4 to 79.3%, with chronic lower extremity injuries being especially common. Lopes et al. (2012) identified that patellofemoral pain syndrome, Achilles tendinopathy, and medial tibial stress syndrome accounted for nearly half of all runners’ RRIs. Ferber et al. (2010) further emphasized the importance of individual physical characteristics, such as hip abductor weakness, in increasing RRI risk, highlighting the need for targeted assessments beyond general training metrics.

To address this need, the Koji Awareness (KA) screening test was developed as a simple, athlete-centered tool for assessing mobility, stability, and postural control to enhance self-awareness and functional monitoring (Murofushi et al., 2022a,2024b; Takasaki and Kanayasu, 2024). Compared with commonly used screening tools, the KA test does not require specialized equipment and can be self-administered without expert evaluation (Murofushi et al., 2022a). A recent prospective study by Murofushi et al. (2025) demonstrated that elite long-distance runners with greater bodily awareness, assessed via the KA test, not only showed significantly greater functional improvements but also remained injury-free throughout the season, although this difference in RRI incidence was not statistically significant. These findings suggest that while bodily awareness contributes to functional adaptation, it may not be sufficient for injury prevention alone.

As Finch (2006) and Donaldson and Finch (2013) have argued, injury prevention strategies are only effective when awareness leads to concrete behavioral modification; this includes consistent implementation of corrective exercises, changes in movement habits, and ongoing self-management informed by functional screening. This awareness–behavior–outcome pathway is also conceptually consistent with research on physical literacy, which suggests that cognitive and perceptual competencies are associated with greater engagement in sport and exercise, thereby contributing to positive health and wellbeing outcomes (Melby et al., 2023). While many studies have separately highlighted the roles of awareness and behavior in sports performance and health, few have directly examined the connection between awareness, behavior change, and subsequent injury risk in real-world competitive settings. From a theoretical perspective, embodied awareness highlights the inseparability of perception and action, as discussed in phenomenology (Merleau-Ponty, 1962) and traditional movement-based practices [***身心一如*** (unity of body and mind)], suggesting that bodily awareness is most meaningful when expressed through observable behavioral change in the training environment.

The study examined the following:

- Relationship between bodily awareness and behavioral change
- Impact of behavioral change on RRI incidence and physical function improvements among elite long-distance runners during a competitive season

We hypothesized that

1. Greater bodily awareness is associated with a higher likelihood of behavioral change.
2. Athletes who reported engaging in behavioral change may demonstrate greater improvements in KA scores and a lower incidence of RRIs compared with those who did not report such changes.

Building explicitly on our previous findings (Murofushi et al., 2025), which identified associations between body awareness and functional outcomes, the present study aims to clarify the mediating role of behavioral change in translating awareness into injury resilience.

## 2 Materials and methods

### 2.1 Participants

This study involved 80 male collegiate long-distance runners belonging to an Ekiden team that regularly competes in the All-Japan University Ekiden Championships, a long-distance relay race among universities. Participants who met any of the following criteria were excluded: (i) severe psychiatric, neurological, or cardiovascular disorders, (ii) current orthopedic conditions with traumatic injuries within 3 months, or (iii) acute infections. Before participation, all runners completed questionnaires on demographic data (age, height, and weight), medical history, athletic background, competitive level, and daily activity levels. Written informed consent was obtained from all participants, and assessments were conducted only after the consent procedures had been completed. Participants were instructed to discontinue the tests immediately if they experienced any pain or discomfort.

This study was approved by the Ethics Committee of Tokyo Science University (approval code: M2019-168) and was conducted in accordance with the Declaration of Helsinki (52nd WMA General Assembly, Edinburgh, 2000).

### 2.2 Protocol

The participants’ attributes, including age, height, weight, and body mass index (BMI), were recorded. All participants underwent the KA screening test 6 months before the Hakone Ekiden season (pre-season). Personalized corrective exercises were prescribed based on the individual results of the KA test; however, although these corrective exercises were included as part of routine practice, they were not implemented or controlled as an experimental intervention, and this study was designed as an observational study. KA testing was repeated after the competitive season (post-season), and the participants completed questionnaires assessing their body awareness and behavioral changes. The team’s dedicated athletic trainer monitored injury incidence and training attendance throughout the 6-month observation period.

### 2.3 Evaluation of awareness and group classification

Participants were asked to rate their body awareness in response to the KA test using a 5-point Likert scale: “Strongly noticed,” “Noticed,” “No change,” “Did not notice,” and “Did not notice at all.” Those who responded with “Noticed” or “Strongly noticed” were classified as the *Awareness group*, and all others were classified as the *Non-awareness group*.

### 2.4 Behavior change evaluation and group classification

Participants also reported changes in their conditioning behaviors based on the KA test by responding to the following question: “Since undergoing the KA screening, how often have you addressed your weak areas in training as self-conditioning?” In this study, behavioral change was defined as athletes’ voluntary and self-initiated engagement in self-conditioning practices, rather than participation in supervised corrective exercises. Accordingly, behavioral change was treated as a qualitative cognitive–behavioral indicator, not as a quantitative intervention effect. Responses were rated on a 5-point Likert scale as follows: “Increased significantly,” “Increased,” “No change,” “Decreased,” and “Decreased significantly.” Those who answered “Increased” or “Increased significantly” were categorized as the *behavioral change group*, while the others were categorized as the *Non-behavioral change group*.

### 2.5 Definition and classification of running-related injuries (RRIs)

In this study, a RRI was defined as a musculoskeletal injury or trauma to the lower extremity or trunk that was triggered by running, resulting in at least 3 consecutive weeks of absence from training or competition (Van Gent et al., 2007; Murofushi et al., 2024b; Kakouris et al., 2021; Francis et al., 2019; Mucha et al., 2017; Lambert et al., 2022; Akoto et al., 2018).

### 2.6 Koji awareness (KA) test

The KA test consists of 11 items that assess mobility, stability, strength, and balance without the need for specialized equipment (Murofushi et al., 2023; Takasaki and Kanayasu, 2024; Murofushi et al., 2022a; Murofushi et al., 2022b; Murofushi et al., 2024a; Murofushi et al., 2024b).

The scoring system is structured according to segment:

- Neck-Scapula-Upper Extremity (NSU) segment: neck mobility, shoulder mobility, scapular mobility, and upper extremity stability/strength (maximum 14 points).
- Trunk segment: thoracic spine mobility, hip and spine mobility, upper/lower extremity mobility and stability, and midsection strength (maximum of 22 points).
- Lower extremity (LE) segments: hip mobility, hip and spine mobility, lower extremity strength, and ankle mobility (maximum 26 points) (Murofushi et al., 2024a).

The total KA scores ranged from 0 to 50. The segmental and total scores were recorded before and after each season. The absolute and percentage changes were calculated based on these results. The KA test has demonstrated high test–retest reliability (Takasaki and Kanayasu, 2024), with an intraclass correlation coefficient (1.1) of 0.876 [95% confidence interval (CI), 0.434–0.981] (Murofushi et al., 2023).

### 2.7 Corrective exercise intervention

Individualized corrective exercises were provided to all participants based on their KA test results. Each KA item corresponds to a specific exercise (Murofushi et al., 2023). Exercises targeting impaired segments were performed three times per week and consisted of three sets of eight repetitions. The exercises were prescribed by an American College of Sports Medicine Certified Exercise Physiologist and performed as part of the warm-up routine of the team under the supervision of the coaches, with exercise intensity and frequency standardized and not individually adjusted or progressed during the study period. Adherence to the corrective exercises was not objectively monitored or quantified, as they were implemented as part of routine practice within this observational study, and exercise intensity and frequency were not individually modified beyond the standardized protocol.

### 2.8 Statistical analysis

Data normality was assessed using histograms and the Shapiro–Wilk test. Descriptive statistics are presented as the mean ± standard deviation for normally distributed variables and as median (interquartile range) for non-normally distributed variables.

Group differences in demographic characteristics were analyzed using unpaired *t*-tests or Mann–Whitney U tests. Associations between group classification (awareness and behavioral change) and occurrence of RRI were assessed using the Fisher’s exact test to analyze the relationship between awareness and behavioral change.

Between-group differences in KA scores and the absolute and percentage changes in scores were analyzed using unpaired *t*-tests or Mann–Whitney U tests, depending on the data distribution. Effect sizes (Fisher’s exact test = Cramer’s V, *t*-test = Cohen’s d, Mann–Whitney U test = r) were calculated for each variable. Effect size thresholds were defined as follows: Cramer’s V values of 0.1, 0.3, and 0.5; Cohen’s d values of 0.2, 0.5, and 0.8; and Mann–Whitney U test r values of 0.1, 0.3, and 0.5, corresponding to small, medium, and large effects, respectively (Fritz et al., 2012).

All statistical analyses were performed using the Statistical Package for the Social Sciences (version 27; IBM Corp., Armonk, NY, United States), and the significance level was set at *p* < 0.05.

## 3 Results

**FIGURE 1:**
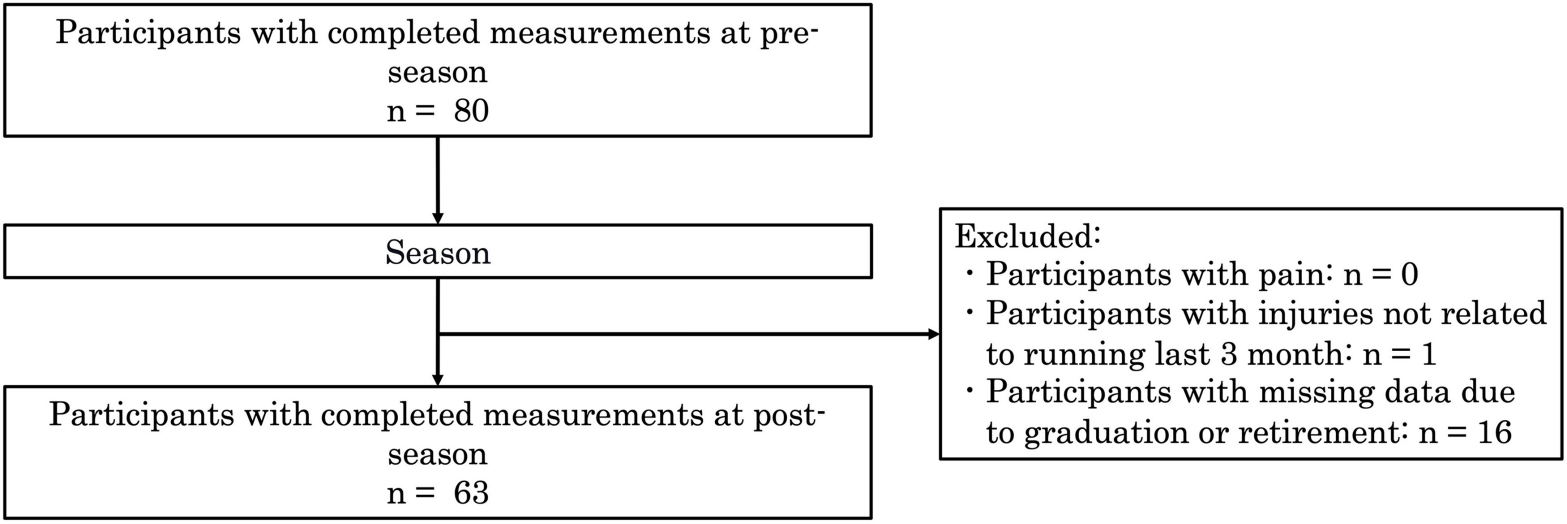
Flow diagram of participant enrollment, exclusions, and retention.

Of the 80 eligible participants, 63 runners were included in the final analysis (Figure 1). Among them, 56 runners (88.9%) were categorized into the Awareness group, having demonstrated a positive awareness response to the KA screening, and seven runners (11.1%) were categorized into the Non-awareness group. Meanwhile, 41 runners (65.1%) were classified into the Behavioral change group, while 22 runners (34.9%) showed no behavioral change. During the 6-month observation period, seven runners sustained running-related injuries (RRIs); however, one injury not related to running was excluded from the final analysis (Figure 1), resulting in seven injuries included in the group comparisons.

There were no statistically significant differences in baseline characteristics between the Awareness and Non-awareness groups, nor between the Behavioral change and Non-behavioral change groups (Table 1).

**TABLE 1:** Demographic data of participants.

Status	Non-awareness group (*n* = 7)	Awareness group (*n* = 56)	*P*-Value	Non-behavioral change group (*n* = 22)	Behavioral change group (*n* = 41)	*P*-value	All
Male, n	7	56	–	22	41	–	63
Age, years^※^	19.0 (1.0)	20.0 (2.0)	0.484	20.0 (2.8)	20.0 (2.0)	0.572	20.0 (2.0)
Height, cm^※^	169.0 (5.0)	172.0 (5.3)	0.354	170.0 (6.0)	172.0 (8.0)	0.156	172.0 (6.5)
Weight, kg	54.4 ± 3.9	56.0 ± 5.3	0.706	54.9 ± 3.8	56.6 ± 4.1	0.219	56.0 ± 4.0
BMI, kg/m^2^	18.9 ± 0.9	18.9 ± 0.8	0.512	18.8 ± 0.8	19.0 ± 0.8	0.558	18.9 ± 0.8

BMI, Body mass index.

^※^Data are represented as the median (interquartile range).

The overall incidence of RRIs during the observation period was 11.1% (Table 2). None of the injuries required surgical intervention, and the types of RRIs sustained and the number of days lost from training or competition are summarized in Table 3. Given the small number of injury cases (*n* = 7), comparisons involving injury outcomes should be interpreted with caution.

**TABLE 2:** Overall occurrence of RRIs during the observation period.

Parameter	Value
Occurrence of injury, n	7
Rate of injury, %	11.1

**TABLE 3:** Types of running related injuries and competition and training time lost in each group.

Type of running injury	Number	Competition and training time lost, days
Plica syndrome	1	42.0
Medial tibial stress syndrome	1	43.0
Iliotibial band friction syndrome	1	37.0
Fibula stress fracture	1	27.0
Achilles tendinitis	2	64.0–78.0
Plantar fasciitis	1	26.0
All	7	

When stratified by level of awareness, the incidence of RRIs was 12.5% in the Awareness group and 0% in the Non-awareness group (Table 4). However, no significant association was found between awareness status and RRI incidence.

**TABLE 4:** Relationship between the degree of awareness and the occurrence of running injuries.

Injury/non-injury	Non-awareness group (*n* = 7)	Awareness group (*n* = 56)	All	*P*-Value
Injury, N(%)	0 (0.0)	7 (12.5)	7 (11.1)	0.419
Non-injury, N(%)	7 (100)	49 (87.5)	56 (88.9)	
All, N (%)	7 (100)	56 (100)	63 (100)	

In contrast, when stratified by behavioral change, the incidence of RRIs was 4.9% in the Behavioral change group, compared to 22.7% in the Non-behavioral change group. A significant association was observed between behavioral change and RRI occurrence (*p* = 0.045, Cramer’s *V* = 0.27; Table 5).

**TABLE 5:** Relationship between the presence or absence of behavior change and the incidence of running injuries.

Injury/non-injury	Non-behavioral change group (*n* = 22)	Behavioral change group (*n* = 41)	All	*P*-Value
Injury, N(%)	5 (22.7)	2 (4.9)	7 (11.1)	0.045
Non-injury, N(%)	17 (77.3)	39 (95.1)	56 (88.9)	
All, N (%)	22 (100)	41 (100)	63 (100)	

**FIGURE 2:**
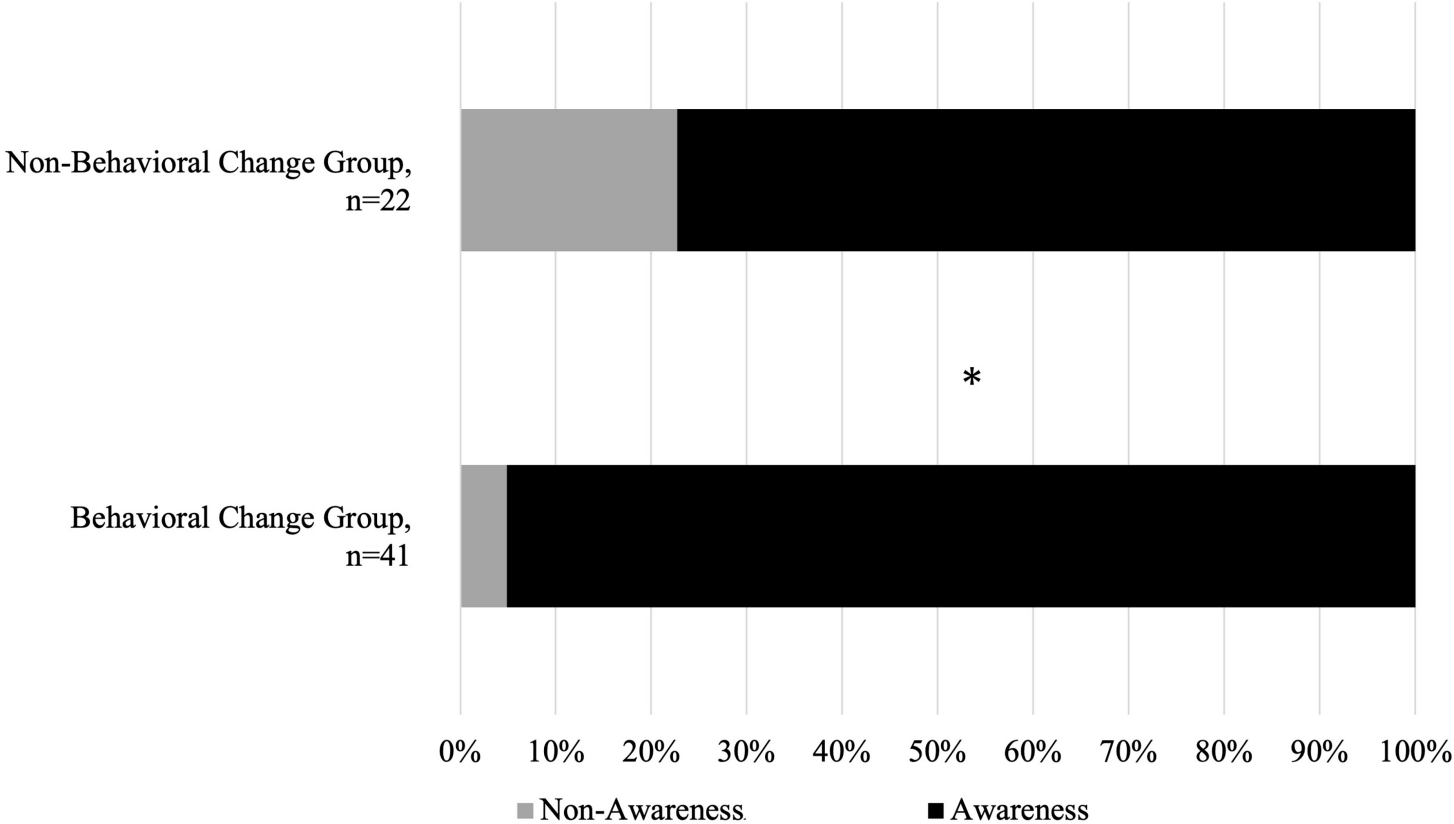
Relationship between awareness and behavioral change. *p = 0.045.

Among those who demonstrated behavioral change, 39 runners (95.1%) belonged to the Awareness group and two (4.9%) to the Non-awareness group (Figure 2). Conversely, among those who did not exhibit behavioral change, 17 runners (77.3%) belonged to the Awareness group and five (22.7%) to the Non-awareness group (Figure 2). A significant association was found between awareness status and behavioral change (*p* = 0.045, Cramer’s *V* = 0.27).

**FIGURE 3:**
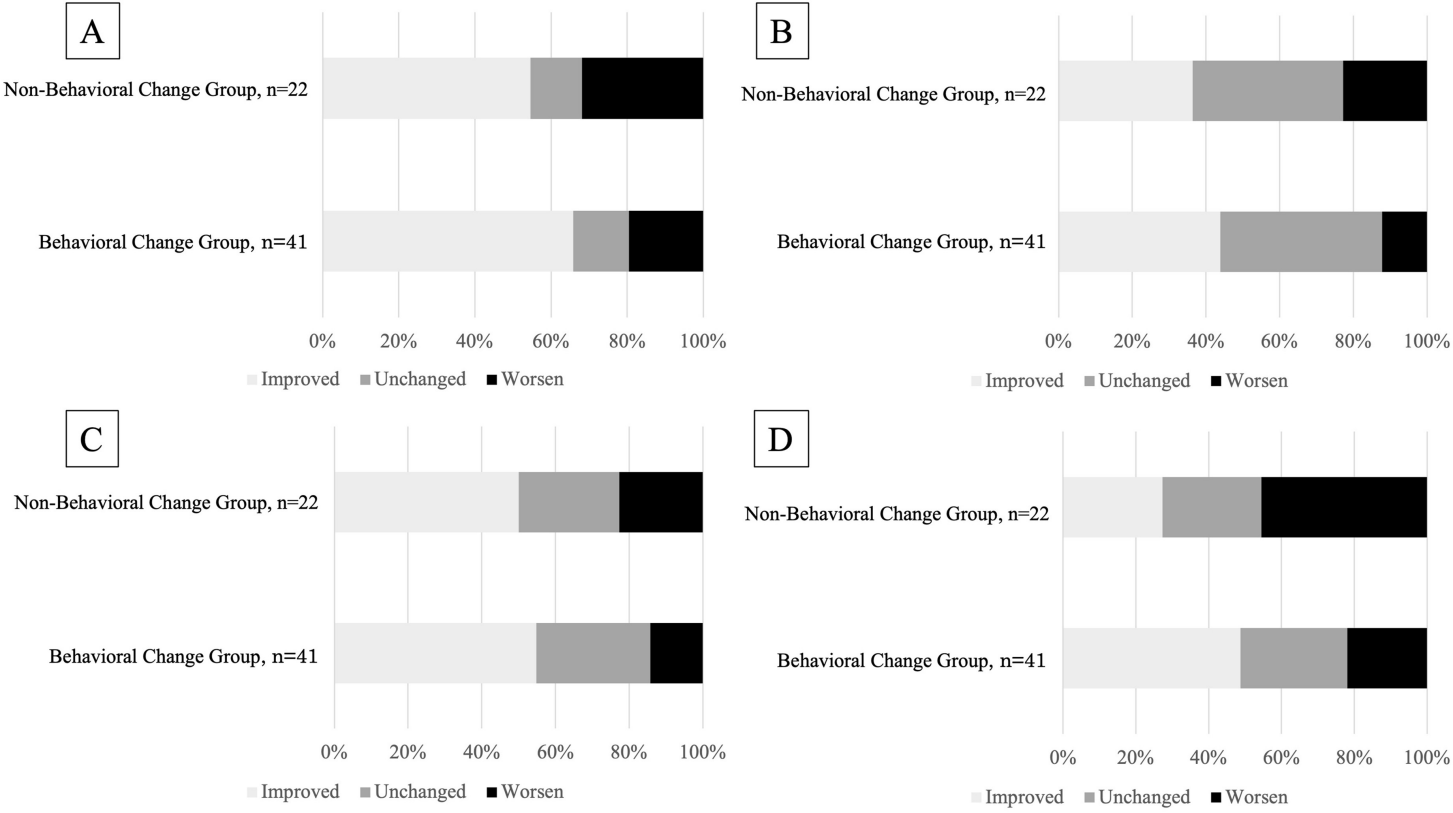
Relationship between the presence or absence of behavioral change and the percentage of people with changes in scores for KA and each sub-item. **(A)** KA total score. **(B)** NSU score. **(C)** Trunk score. **(D)** LE score. KA, Koji Awareness; NSU, Neck scapula-upper extremity; LE, Lower extremity.

In terms of lower extremity (LE) scores prior to the intervention (pre-season), the Behavioral change group had significantly lower baseline LE scores compared to the Non-behavioral change group (*p* = 0.009, *r* = −0.33; Table 6). This baseline difference suggests the presence of potential confounding, as participants with poorer initial function may have been more likely to engage in behavioral change. However, both the amount and percentage of improvement in LE scores during the follow-up period were significantly greater in the Behavioral change group (*p* = 0.33, *r* = −0.27; Table 6). No significant differences were found for other subscale scores. The relationship between behavioral change and the proportion of participants showing improvement, no change, or deterioration in KA total and sub-item scores is illustrated in Figure 3.

**TABLE 6:** Differences between groups in scores and change rate in KA ^※^.

Parameter	Non- behavioral change group (*n* = 22)				Behavioral change group (*n* = 41)			
	Pre	Post	Amount of change	Rate of change	Pre	Post	Amount of change	Rate of change
Total	45.0 ± 4.8	45.5 ± 3.2	0.5 ± 4.1	2.0 ± 10.4	43.4 ± 5.0	45.7 ± 3.3	2.3 ± 3.9	6.3 ± 11.2
UE part	12.5 ± 1.6	13.0 ± 1.3	0.5 ± 1.7	3.2 ± 12.0	12.5 ± 1.6	13.1 ± 1.5	0.6 ± 1.7	4.2 ± 12.5
Trunk part	20.0 ± 2.4	20.4 ± 2.0	0.4 ± 2.2	2.1 ± 9.9	19.5 ± 2.6	20.7 ± 1.6	1.2 ± 2.2	5.4 ± 10.2
LE part	24.1 ± 2.8	23.7 ± 1.7	−0.4 ± 3.0	−1.4 ± 11.4	22.4 ± 3.2^*^	23.4 ± 2.1	1.0 ± 2.6^**^	3.9 ± 9.8 ^***^

^※^Data are represented as the average ± standard deviation. *p = 0.009 (compared to the Non-behavioral change group). **p = 0.033 (compared to the Non-behavioral change group). ***p = 0.033 (compared to the Non-behavioral change group). KA, Koji Awareness; NSU, Neck scapula-upper extremity; LE, Lower extremity.

## 4 Discussion

In this study, we used the KA screening test to explore the relationships between bodily awareness, behavioral change, and injury incidence in elite collegiate long-distance runners. While most runners (88.9%) reported heightened bodily awareness, only behavioral change, not awareness alone, was significantly associated with a lower risk of RRIs. Moreover, runners who exhibited behavioral changes also showed greater lower extremity function improvement during the season, despite having lower baseline scores. These findings support our hypothesis that awareness alone may be insufficient for injury prevention and that behavioral change is associated with both injury reduction and functional recovery.

Although prior research (Murofushi et al., 2025) suggested that bodily awareness may be associated with functional improvements and possibly injury resilience, the present findings indicate that awareness by itself does not significantly reduce injury incidence. This interpretation is based on observed associations and should not be understood as evidence of a direct causal relationship. This is conceptually consistent with theoretical models such as the Health Belief Model (Rosenstock, 1974) and Transtheoretical Model (Prochaska and Velicer, 1997), which suggest that awareness of risk may need to be accompanied by factors self-efficacy, motivation, and readiness to change to result in behavioral outcomes. It should be noted that these psychological constructs were not directly measured in the present study and are therefore discussed here as speculative explanatory frameworks. Most runners in our study recognized their physical issues, but only those who converted that awareness into action experienced significantly fewer injuries.

Runners who reported modifying their self-conditioning habits, based on perceived physical weaknesses, experienced a significantly lower injury rate (4.9% vs. 22.7%). This finding indicates an association between self-reported behavioral change and injury incidence, supporting Finch’s (2006) argument that injury prevention depends not only on identifying risk factors but also on engaging in targeted actions. Interestingly, those who made behavioral changes began the season with lower LE scores, indicating poorer function, yet improved more than their counterparts. This baseline difference suggests that participants with poorer initial functional status were more likely to engage in behavioral change. Accordingly, the KA test may be useful for identifying individuals at potential risk of RRIs and encouraging them to implement self-conditioning strategies aimed at risk reduction. Moreover, this pattern may suggest that athletes with greater functional deficits experienced heightened bodily awareness and were therefore more likely to engage in corrective behaviors.

Importantly, while various factors such as prior injury history and training load were not accounted for in the analysis, a significant association between bodily awareness and behavioral change was observed in this study. This supports the idea of awareness as a precursor or enabler. From a phenomenological standpoint, Merleau-Ponty (1962) described the body not as an object but as a subject that perceives, acts, and adapts. Zen and Budo philosophies similarly emphasize “**身心一如**” (unity of body and mind), implying that genuine awareness must manifest in action. In this sense, the KA test has the potential to function as a catalyst for embodied self-reflection and purposeful behavior. In addition, the findings of this study suggest that bodily awareness gained through the KA test may be associated with the encouragement or support of behavioral change. Therefore, using the KA test, practitioners may not only assess athletes’ physical condition but also guide them in translating insights into sustainable conditioning habits. The hypothesized sequence in which awareness leads to behavioral change and subsequently relates to injury outcomes was not formally tested (e.g., via mediation analysis) and should therefore be regarded as a conceptual model rather than an empirically demonstrated pathway. These perspectives are presented to aid conceptual interpretation and do not constitute direct empirical evidence within this study.

### 4.1 Limitations

This study has some limitations that should be considered when interpreting the results. First, the study was conducted at a single institution and involved a relatively homogeneous cohort of male collegiate long-distance runners. This homogeneity may introduce selection bias and limit the generalizability of the findings to broader athletic populations, including female athletes and other sports disciplines.

Second, key variables such as awareness and subsequent behavioral change were assessed retrospectively after the competitive season, relying on single-item, non-validated self-report questionnaires. As a result, recall bias, social desirability bias, and individual differences in interpretation may have influenced these subjective measures, thereby reducing measurement precision.

Third, the number of injury events was relatively small, which may have limited the statistical power to detect meaningful differences and increased the risk of type II error. In addition, potential confounding variables (e.g., prior injury history, training load, baseline function, coaching strategies, and psychological characteristics) were not fully controlled, which may have influenced the observed associations.

Fourth, although corrective exercises were implemented in a structured setting, objective data on individual adherence, execution quality, and actual behavioral change were not collected. Furthermore, the corrective exercise intervention itself may have influenced both awareness and functional outcomes, making it difficult to disentangle the independent effects of awareness-related behavioral change.

Future research should adopt a multicenter design with more diverse athletic populations, incorporate validated multidimensional instruments to assess awareness such as MAIA (Mehling et al., 2009, 2012) and transtheoretical model (Liu et al., 2018), and utilize objective measures of behavior and adherence (e.g., digital tracking or wearable sensor data). Additionally, examining mediating and moderating factors such as motivation, coaching influence, and personality traits may help clarify the mechanisms through which awareness translates into sustained injury prevention and functional adaptation.

## 5 Conclusion

This prospective study found an association between behavioral changes following KA screening and a lower incidence of running-related injuries, as well as greater improvements in lower extremity function, in a cohort of elite collegiate male long-distance runners. While bodily awareness was not directly associated with injury incidence, it showed an association with functional improvement and was related to the presence of subsequent behavioral change.

These findings suggest that body awareness may function as a cognitive–perceptual factor that is linked to adaptive behavior, rather than exerting a direct protective effect on injury risk. In this context, the KA test may serve not only as a screening tool but also as a contextual platform through which athletes reflect on bodily sensations and training-related responses, particularly during periods of high training load.

Given the observational nature of this study, these results should be interpreted cautiously and should not be generalized beyond similar athletic populations. Nevertheless KA-based intervention may represent a promising framework for supporting awareness-informed behavioral adaptation by restoring the dialogue between athletes and their bodies, thereby contributing to performance, sustainable injury resilience, and long-term athlete development.

## Data Availability

The raw data supporting the conclusions of this article will be made available by the authors, without undue reservation.
